# Integrating yeast biodiversity and machine learning for predictive metabolic engineering

**DOI:** 10.1093/femsyr/foaf072

**Published:** 2025-12-03

**Authors:** Akaraphol Watcharawipas, Weerawat Runguphan, Peerapat Khamwachirapithak, Thanaporn Laothanachareon

**Affiliations:** Department of Microbiology, Faculty of Science, Mahidol University, 272 Rama VI Road, Ratchathewi, Bangkok 10400, Thailand; National Center for Genetic Engineering and Biotechnology, 113 Thailand Science Park, Phahonyothin Road, Khlong Nueng, Khlong Luang, Pathum Thani 12120, Thailand; Department of Biotechnology, Faculty of Science and Technology, Rangsit Campus, Thammasat University, Phahonyothin Road, Khlong Luang, Pathum Thani 12120, Thailand; National Center for Genetic Engineering and Biotechnology, 113 Thailand Science Park, Phahonyothin Road, Khlong Nueng, Khlong Luang, Pathum Thani 12120, Thailand

**Keywords:** yeast, biodiversity, machine learning, metabolic engineering, synthetic biology, strain development

## Abstract

Yeast biodiversity and machine learning (ML) are transforming the landscape of metabolic engineering. While *Saccharomyces cerevisiae* remains foundational to industrial biotechnology due to its genetic tractability and robust growth, it struggles to synthesize complex metabolites, utilize alternative feedstocks, and withstand industrial stresses. Non-conventional yeasts such as *Yarrowia lipolytica* and *Ogataea polymorpha* possess traits such as thermotolerance, acid resistance, and lipid accumulation, making them promising alternatives. However, broader adoption remains limited by insufficient genetic tools and low predictability of engineered components across species. Recent ML advances are addressing these gaps by enabling accurate prediction of genetic part function, optimizing gene expression, and discovering novel biosynthetic components in diverse yeasts. These tools support rational selection of genetic elements and pathway configurations tailored to non-model hosts, streamlining the design–build–test–learn cycle. Leveraging biodiversity expands the available yeast chassis and toolkits, improving strain robustness under industrial conditions. This mini-review discusses how yeast biodiversity is being harnessed to broaden engineering strategies and highlights recent ML advances driving data-guided strain and pathway design. Special attention is given to ML-guided identification and optimization of genetic elements. Together, evolutionary diversity and intelligent computation promise more modular, predictive, and scalable yeast platforms for next-generation metabolic engineering.

## Teaser

Combining yeast biodiversity with machine learning is a strategy for the next generation of metabolic engineering.

## Introduction

Yeasts have long served as important platforms in biotechnology and metabolic engineering due to their rapid growth, genetic versatility, and ability to perform post-translational modifications. Among them, *Saccharomyces cerevisiae* is the most extensively studied species and has been widely used for the production of ethanol, proteins, and a variety of industrial chemicals. Nevertheless, the growing complexity of industrial demands, such as synthesizing complex natural products, utilizing alternative feedstocks, and operating under harsh fermentation conditions, highlights significant limitations of relying solely on *S. cerevisiae*.

Consequently, there has been increasing interest in exploring non-conventional yeasts such as *Yarrowia lipolytica, Ogataea polymorpha, Kluyveromyces marxianus*, and *Rhodotorula glutinis*. Non-conventional yeasts such as *Y. lipolytica* and *O. polymorpha* exhibit traits such as thermotolerance, acid resistance, and lipid accumulation, though these characteristics vary among species. Yet, widespread industrial adoption is hampered by their underdeveloped toolkits, lower transformation efficiencies, and poorly characterized regulatory elements. While advances like the Yeast MoClo modular cloning platform (Lee et al. 2015) have begun addressing these challenges, reliable predictions of part performance in different yeast species remain elusive.

Parallel developments in synthetic biology increasingly emphasize predictive, data-driven designs. Machine learning (ML), capable of analyzing complex biological datasets, is becoming key to metabolic engineering. Recent ML studies in yeast have successfully predicted promoter activity (Kotopka and Smolke 2020), optimized gene expression (Yan et al. 2024), and designed synthetic regulatory elements (de Boer et al. 2020, Linder et al. 2020). These approaches are greatly enhancing traditional design–build–test–learn (DBTL) cycles.

At the same time, yeast biodiversity represents an underexplored resource for synthetic biology. The genomes and traits of hundreds of yeast species contain diverse biosynthetic genes, regulatory sequences, and stress responses. Pioneering initiatives such as Y1000+ are generating large-scale multi-species datasets, enabling ML-driven genotype-to-phenotype predictions, facilitating cross-species discovery of genetic parts, and expanding host selection (Harrison et al. 2024).

This convergence of ML and biodiversity is poised to shift yeast engineering toward more modular, transferable, and predictive frameworks. However, several challenges remain. Many ML tools are still trained exclusively on *S. cerevisiae*, limiting their generalizability. Non-conventional yeasts often lack standardized annotations and tools for high-throughput experimentation, which are critical for developing robust predictive models. Addressing these issues will require advances in data curation, model interpretability, and cross-species benchmarking.

**Figure 1.**
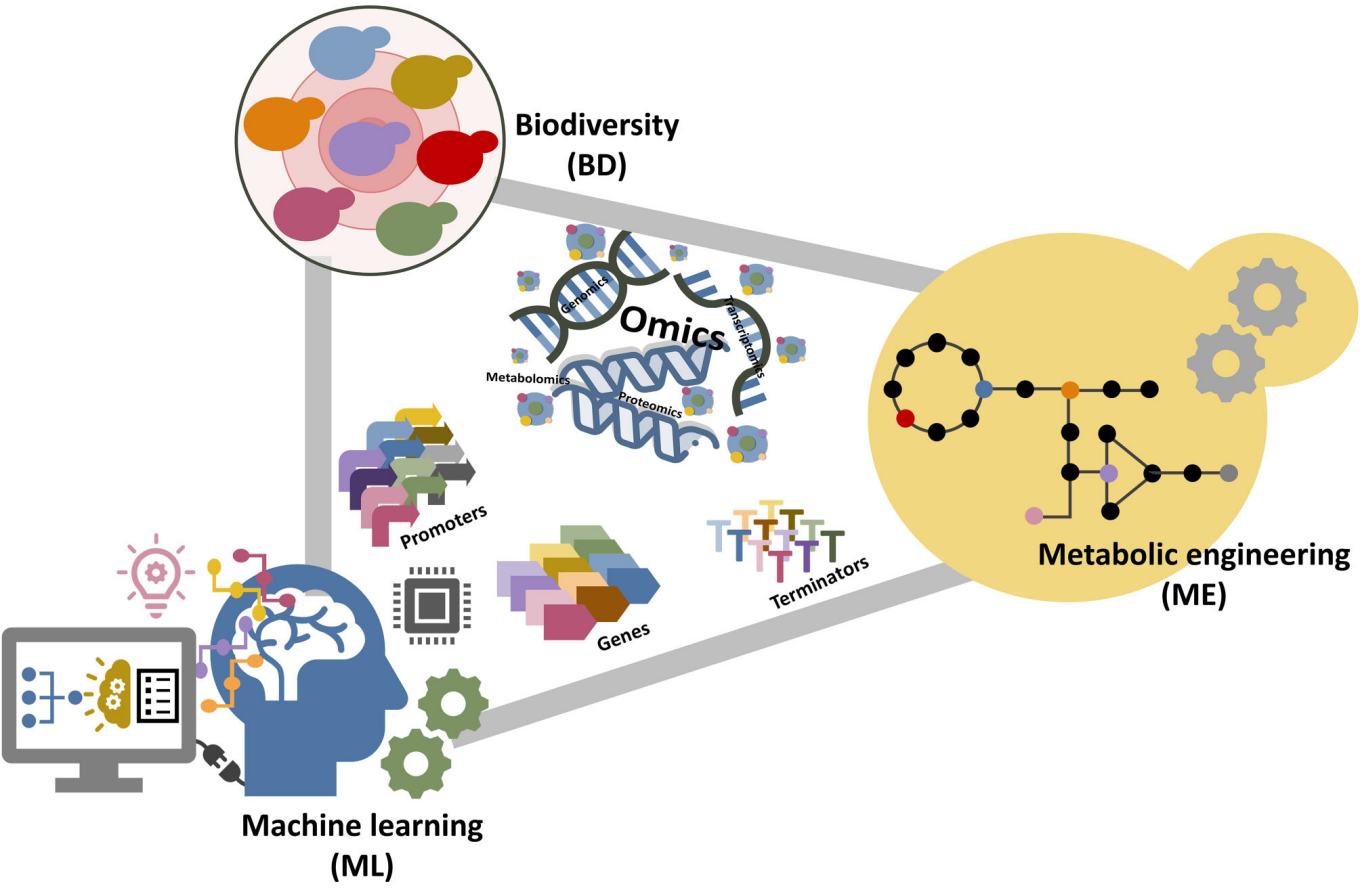
Harnessing the synergy between yeast biodiversity (BD) and machine learning (ML) to drive efficient and predictive metabolic engineering (ME) for improved yeast strain design.

In this mini-review, we begin by highlighting how yeast biodiversity can enrich the genetic toolbox and chassis selection available to metabolic engineers. We then examine recent advances in ML that support strain design, regulatory element optimization, and pathway engineering in yeast. Finally, we propose that effectively integrating biodiversity-informed approaches with ML-guided design will create robust, versatile, and scalable yeast engineering platforms, poised to transform the landscape of synthetic biology (Fig. 1). This review uniquely focuses on the intersection between yeast biodiversity and ML, emphasizing how biodiversity-derived datasets drive the development of predictive models for metabolic engineering. Unlike previous ML-only reviews, this work highlights cross-species generalization of ML frameworks and biodiversity-informed discovery of genetic parts, offering a holistic view of how evolutionary diversity can be computationally harnessed for next-generation yeast design.

## Leveraging yeast biodiversity for metabolic engineering

**Figure 2.**
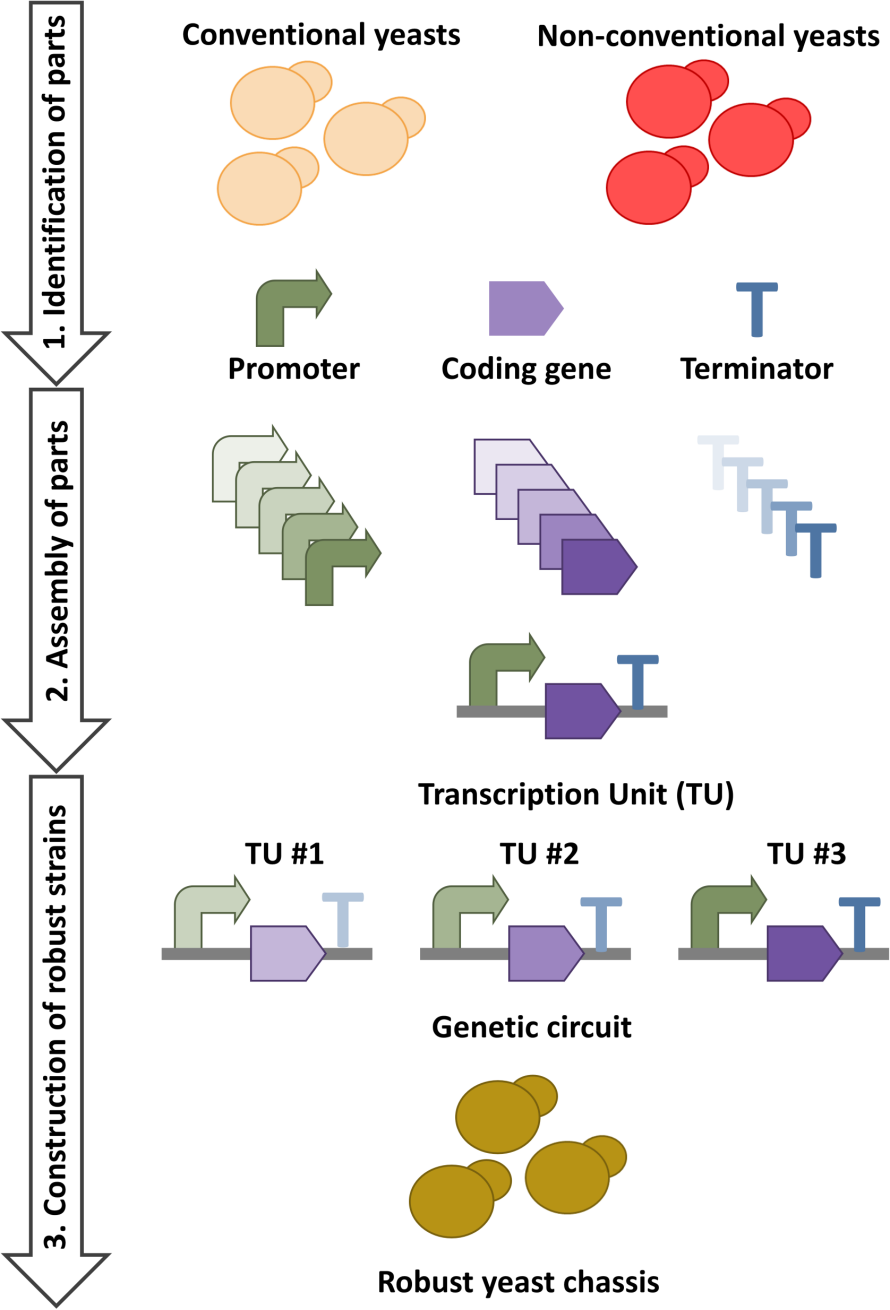
Strain engineering workflow for building robust microbial chassis. The process involves three steps: (1) Identifying genetic parts, including promoters, coding sequences, and terminators, to create standardized components, despite limitations in diversity and performance. (2) Assembling these parts into transcription units and genetic circuits, optimized via combinatorial design and modular cloning to boost productivity. (3) Expressing the engineered circuits in a tailored yeast chassis for robust performance.

Yeast-based synthetic biology is progressively advancing to meet the growing demand for sustainable production of high-value chemicals. A key challenge is building robust microbial platforms that function efficiently across diverse industrial conditions and feedstocks, while achieving commercially viable yields (Chen et al. 2020). Achieving this goal requires several key steps in the workflow (Fig. 2). First, it involves identifying genetic elements such as promoters, genes, and terminators. These elements can be natural or synthetic, with the main bottleneck being their diversity, utility, and efficiency (Decoene et al. 2018). Second, these parts are assembled into transcription units and combined into genetic circuits. Optimizing genetic circuits is challenging but can be addressed through combinatorial design and modular cloning techniques (Naseri and Koffas 2020). Third, optimized genetic circuits must function effectively within suitable host systems, raising questions about ideal chassis selection and customization (Dixon and Pretorius 2020). Based on this workflow, yeast biodiversity is promising for discovering new efficient regulatory elements and novel gene variants for expanding the repertoire of microbial chassis available for metabolic engineering.

Yeasts are unicellular fungi reproducing asexually by budding or fission, and sexually via spores without forming fruiting bodies (Kurtzman et al. 2011). They belong primarily to the phyla Ascomycota, which includes the subphyla Saccharomycotina (budding yeasts) and Taphrinomycotina (fission yeasts), and Basidiomycota, comprising the subphyla Ustilaginomycotina, Pucciniomycotina, and Agaricomycotina (Li et al. 2021b). Yeasts play critical roles in organic matter recycling across diverse environments, including tropical forests (Rosa et al. 2023), peat swamp forests (Satianpakiranakorn et al. 2020), and marine habitats (Kaewkrajay et al. 2020). Recently, Boekhout et al. showed that most new yeast species discoveries (63%) occurred in tropical and subtropical regions, predominantly Asia, highlighting geographic gaps in exploration elsewhere (Boekhout et al. 2022).

Yeast biodiversity, along with comprehensive taxonomic information, is now accessible via The Yeasts Trust Database (https://theyeasts.org/). Several studies highlight its biotechnological potential. For instance, Aravind *et al*. isolated *Cryptococcus flavescens* SK01 from an endangered tree in India, demonstrating high production (4.7 g/l) of exopolysaccharides (EPS) such as mannans, phosphomannans, galactomannans, glucomannans, and glucuronoxylomannans (Aravind et al. 2014). Grondin et al. isolated 101 yeast strains from tropical fruits in the South-West Indian Ocean, classifying 26 distinct species via large-subunit rRNA sequencing (Grondin et al. 2015). Two isolates, from Cape gooseberry and cocoa beans, shared 97.1% and 97.4% sequence similarity with *Rhodotorula mucilaginosa* and *Candida pararugosa*, respectively—values below the typical 98.4%–99% similarity threshold (Kurtzman and Robnett 1998) used for species delimitation in yeasts—thereby suggesting the presence of novel taxa. They also identified 52 volatile organic compounds (VOCs), including six rare markers for specific species, and statistical analysis linked each species to a distinct aroma profile, with *Saprochaete suaveolens* producing the most diverse VOC profile. Similarly, Jaiboon et al. isolated 65 ethanol- and indole-3-acetic acid (IAA)-producing yeasts from To Daeng peat swamp forest, in Thailand, showcasing diversity in metabolic capabilities (Jaiboon et al. 2016). All produced ethanol from glucose, ranging from 9.0–58.0 g/l, with *Cyberlindnera subsufficiens* DMKU-YNB42-1 yielding the highest yield, whereas 62 strains produced IAA between 9.0 and 66.9 mg/l, with the highest titer is from *Rhodotorula mucilaginosa* DMKU-Y33-A. Nearly 200 unidentified basidiomycetous yeasts were collected from plant surfaces and soil, resulting in the identification of 107 new species, including *Tremella*, known for producing EPS with therapeutic properties such as *Tremella aurantialba* polysaccharide B1, a heteropolysaccharide consisting of D-mannose, D-xylose, and D-glucuronic acid in a molar ratio of 3.1:2.9:1.2, and its acetylated derivative *Tremella aurantialba* polysaccharide A1 (Li et al. 2020a, Rahbar Saadat et al. 2021). Shi et al. (2020) identified cold- and metal-tolerant glutathione S-transferase from an Antarctic yeast, *Rhodotorula mucilaginosa* AN5, showing stability under high salt, acidity, and metal stress, suggesting adaptive metabolisms. Phuengjayaem et al. (2023) isolated astaxanthin-producing yeasts from fruits and flowers across Thailand, with *R. paludigena* strain SP9-15 yielding high astaxanthin levels at 6.565 ± 0.238 mg/l. Whole-genome sequencing revealed carotenogenic genes (*CrtE, CrtYB, CrtI, CrtS, CrtW, CrtZ*), potentially representing novel enzyme variants for carotenoid synthesis. Similarly, our group identified high-performance carotenoid biosynthetic genes from 26 Thai red yeasts, revealing that the carotenogenic genes *CrtE, CrtYB*, and *CrtI* from *Sporidiobolus pararoseus* TBRC-BCC 63403 outperformed benchmark genes from the red yeast *Xanthophyllomyces dendrorhous* in beta-carotene production (Watcharawipas et al. 2021). These studies collectively underscore the potential of yeast biodiversity to provide valuable genetic resources for metabolic engineering.

Yeasts can be categorized into conventional and non-conventional groups based on their research and industrial application histories. Conventional yeasts including the budding yeast *S. cerevisiae* and the fission yeast *Schizosaccharomyces pombe* are well-characterized and extensively used as model organisms (Naranjo-Ortiz and Gabaldón 2019). However, *S. cerevisiae* has limitations such as restricted substrate utilization, sensitivity to stress conditions (e.g. high temperature and low pH), and a metabolic bias toward ethanol production (the Crabtree effect), which diverts carbon flux away from target metabolites (Li et al. 2022a). Under high-carbon conditions, this Crabtree effect becomes a major industrial bottleneck, necessitating metabolic rewiring or the use of alternative yeast hosts. Consequently, non-conventional yeasts have emerged as promising candidates due to unique traits beneficial for industrial processes (Table 1). The following section highlights some noteworthy non-conventional yeast species.

**Table 1.** Comparison of major traits of non-conventional yeasts with industrial relevance.

Yeast species	Natural habitat/origin	Stress tolerance	Carbon utilization	Industrial relevance	References
*Komagataella phaffii* (formerly *Pichia pastoris*)	Tree exudates and slime fluxes from oak or chestnut trees	Moderate thermotolerance (≤32°C); sensitive to high methanol/formaldehyde stress	Methanol, glycerol, glucose, sorbitol	Widely used for recombinant protein, enzyme, and organic acid production	Karbalaei et al. (2020); Bernauer et al. (2021); Wang et al. (2024);
*Ogataea polymorpha*	Spoiled fruits, soil, plant exudates	Thermotolerant up to 48°C; relatively tolerant to methanol and oxidative stress	Methanol, glycerol, glucose, xylose	Thermotolerant chassis for fatty acids, lactate, sesquiterpenoids	Thorwall et al. (2020); Gao et al. (2022); Wefelmeier et al. (2023)
*Yarrowia lipolytica*	Oil-rich environments, dairy and meat products	pH- and salt-tolerant; robust under nutrient limitation	Fatty acids, glycerol, alkanes, glucose	Lipid and organic acid bioproduction; platform for acetyl-CoA derivatives	Lopes et al. (2022); Park and Ledesma-Amaro (2023); Shen et al. (2024)
*Rhodotorula mucilaginosa*	Saline, hydrocarbon-contaminated, or plant-associated habitats	Highly tolerant to osmotic and oxidative stress; resistant to inhibitors	Glucose, glycerol, hydrocarbons	Carotenoids, terpenoids, and single-cell oil production	Leyton et al. (2019); Prabhu et al. (2019); Benmessaoud et al. (2022); Chen et al. (2024)

*Komagataella phaffii* (formerly *Pichia pastoris*) is a methylotrophic yeast first isolated from the exudate of a chestnut tree in France in 1919 (Bernauer et al. 2021). It supports high-density growth, glycosylation, methanol-inducible expression via the strong AOX1 promoter, and efficient protein secretion (Karbalaei et al. 2020, Eskandari et al. 2023, Muzaffar et al. 2025). Recent genetic toolkits, including CRISPR/Cas9 systems (Yang et al. 2020), synthetic promoter libraries (Dou et al. 2021, Liu et al. 2024), and genome-scale metabolic models (Subash Chandra Bose et al. 2023) have transformed it from a protein expression host into a versatile microbial chassis for the production of not only recombinant proteins and enzymes, but also industrial biochemicals (Pan et al. 2022, Vijayakumar and Venkataraman 2024, Moon et al. 2025). Although not universally granted GRAS status, it is approved in food and feed applications on a case-by-case basis. However, limitations include methanol toxicity and accumulation of formaldehyde, which impair cell viability (Wang et al. 2024), and challenges in deploying human-compatible post-translational modifications, necessitating glycoengineering, in certain biopharmaceutical applications.

*Ogataea polymorpha*, another methylotrophic yeast, is found in various natural niches such as spoiled fruits and soil (Xie et al. 2024). It demonstrates thermotolerance up to 48°C (Thorwall et al. 2020), broad carbon utilization, including methanol, glycerol, glucose, and xylose (Li et al. 2022d), and human-compatible glycosylation (Manfrão-Netto et al. 2019). Genetic toolkits, including plasmids (Bratiichuk et al. 2020), regulatory elements (promoters and terminators) (Zhai et al. 2021, Wefelmeier et al. 2022, Yan et al. 2022), and genome editing via CRISPR/Cas9 (Gao et al. 2021) and Cas12a (Hou et al. 2025), have enhanced strain engineering, enabling production of sesquiterpenoids like β-elemene (Ye et al. 2023). Adaptive laboratory evolution (ALE) has enabled improved growth and titers of free fatty acid (up to 15.9 g/l) and lactate (3.8 g/l) following genetic manipulations (Gao et al. 2022, Wefelmeier et al. 2023). A genome-scale model has also supported rewiring metabolism toward the production of malate, acetone, and isoprene (Wefelmeier et al. 2024). Despite its attractive features, *O. polymorpha* faces key challenges, including compromised methanol utilization (Zhai et al. 2023), the fact that homologous recombination (HR) enhancement can impair robustness (Jia et al. 2025), and incomplete genome annotation still hinders optimization.

*Yarrowia lipolytica* naturally inhabits oil-rich environments and thrives across diverse pH and temperatures (Lopes et al. 2022, Park and Ledesma-Amaro 2023). It metabolizes fatty acids, vegetable oils, and animal fats as energy sources (Jach and Malm 2022), and secretes industrial enzymes like lipase, protease, and glucosidase, useful in food and pharmaceutical industries (Guardiola et al. 2021). It can be engineered using CRISPR/Cas9 and modular cloning methods like Golden Gate assembly (Cui et al. 2021, Cao et al. 2023). For example, the YALIcloneNHEJ toolkit enables efficient random multigene integration via non-homologous end-joining (NHEJ) (Li et al. 2022c), while YALIcloneHR expanded this capability to targeted integration using homologous arms and CRISPR/Cas9. The system was validated through *PEX10* knockout and successfully applied to arachidonic acid production, achieving a yield of 4.8%, demonstrating its potential for acetyl-CoA-derived chemical production (Shen et al. 2024). Although methanol is a sustainable feedstock, *Y. lipolytica* cannot naturally utilize it efficiently. Jiang et al. recently engineered methanol assimilation in *Y. lipolytica* using the RuMP and XuMP pathways from *Bacillus methanolicus* and *P. pastoris*, enabling methanol-supported resveratrol biosynthesis (Jiang et al. 2025). These cross-species designs illustrate the potential of biodiversity-guided engineering via genetic transfer across species.

*Rhodotorula mucilaginosa*, a red basidiomycete yeast, is found in saline and hydrocarbon-contaminated habitats (Li et al. 2020b, Leyton et al. 2019, Benmessaoud et al. 2022). It naturally accumulates terpenoids and >40% lipids (Li et al. 2022e). Its high tolerance to hyperosmotic stress and resistance to lignocellulosic inhibitors make *R. mucilaginosa* a promising chassis for producing carotenoids and single-cell oils (Prabhu et al. 2019). Chen et al. (2024) enhanced the mevalonate (MVA) pathway of *R. mucilaginosa* strain JY1105 and expressing *Vitis vinifera* α-terpineol synthase, resulting in α-terpineol production at 0.39 mg/l. Overexpressing α-farnesene synthase from *Malus domestica* and β-ionone synthase from *Petunia hybrida* led to 822 mg/l α-farnesene and 0.87 mg/l β-ionone, respectively. Despite its potential, *R. mucilaginosa* faces challenges due to a poorly annotated genome and limited genetic tools. Progress in comparative genomics and synthetic biology toolkits, often adapted from the closely related and better-studied *Rhodosporidium toruloides*, is expected to enhance its utility (Nora et al. 2019, Otoupal et al. 2019, Wen et al. 2020).

Biodiversity-driven approaches such as heterologous expression of biosynthetic pathways from diverse species, comparative genomics for part discovery, and ALE represent powerful strategies for unlocking the potential of yeast biodiversity. The vast, underexplored yeast biodiversity provides a valuable resource of novel promoters, terminators, genes, and even transcription factors (TFs) for integrating into synthetic biology workflows. However, challenges persist, notably limited genetic tools, incomplete genome annotations, and the difficulty of domesticating non-model yeasts. Overcoming these barriers through ML-assisted omics analyses, advanced genome-editing technologies, and genome-scale metabolic modeling will be critical for realizing the full potential of yeast biodiversity.

## Advances in ML for metabolic engineering

**Figure 3.**
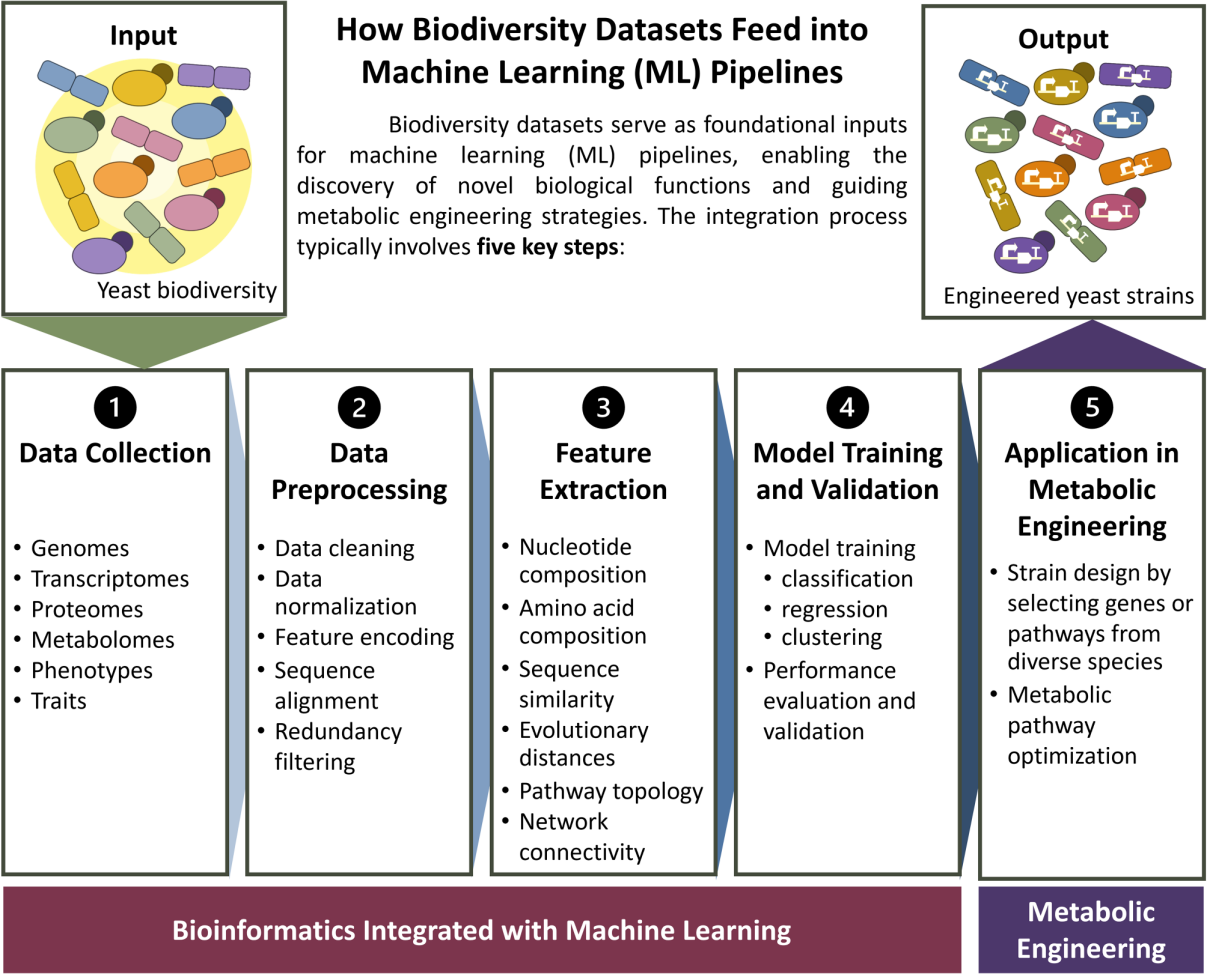
Machine learning pipeline integrating biodiversity datasets to enable the discovery of novel biological functions and guide metabolic engineering strategies. The pipeline comprises five key steps: (1) Data Collection, where biodiversity-related genomic, phenotypic, and environmental data are gathered; (2) Data Preprocessing, involving data cleaning, normalization, and integration; (3) Feature Extraction, to identify relevant biological descriptors and construct meaningful representations; (4) Model Training and Validation, where predictive or generative ML models are developed and evaluated; and (5) Application in Metabolic Engineering, in which model-derived insights inform the design of strains, pathway optimization, and functional characterization.

The expanding catalog of yeast genomes and phenotypes now serves as a valuable resource for ML model training. Sequence, transcriptomic, and metabolic data obtained from biodiversity exploration provide high-dimensional datasets that feed directly into ML pipelines (Fig. 3). These data enable the prediction of promoter strength, enzyme activity, and pathway performance across diverse yeast species. Integrating biodiversity-derived datasets into ML frameworks thus represents a crucial link between natural diversity and predictive design.

Recent advances in metabolic engineering have increasingly leveraged artificial intelligence, particularly ML. ML has demonstrated great potential computational methods for classification and regression in metabolic engineering, strain optimization, and bioprocess development. Integrated into metabolic engineering and synthetic biology workflows, ML enhances the DBTL cycle. ML methods can be categorized into two main categories: supervised learning, which requires labeled data as input and a corresponding output, and unsupervised learning, which exploits unlabeled data and identifies useful patterns or relationships. Both supervised and unsupervised ML methods generally do not require prior knowledge of the mechanisms or biological functions.

Classical supervised learning methods comprise linear and logistic regression, support vector machine (SVM), random forest (RF), and gradient boosting, each suited for specific classification or regression tasks. Supervised learning predicts numeric outputs from input data or categorizes input data into specific groups using labeled input-output data. In contrast, unsupervised learning identifies significant patterns in unlabeled data, commonly applied to clustering and dimensionality reduction in biological datasets. Typical algorithms, including k-means clustering, hierarchical clustering, principal component analysis, and t-distributed stochastic neighbor embedding (t-SNE), are common unsupervised learning algorithms for clustering and dimensionality reduction in omics data (Greener et al. 2022). Additionally, deep learning, driven by expanding biological data from high-throughput techniques, employs artificial neural networks (ANNs) that mimic brain-like interconnected nodes. Deep learning can solve more complex problems than conventional ML methods.

In yeast metabolic engineering, ML has been integrated into the iterative DBTL cycle to optimize pathways, strains, and bioproduction, as well as predict genotype–phenotype relationships. A notable application is the Automated Recommendation Tool (ART), which uses Bayesian optimization to predict high-performing strain designs based on prior DBTL data (Radivojević et al. 2020). ART leverages experimental data, for instance, gene and protein expression levels linked to bioproduction levels, and employs Bayesian ensemble and Markov Chain Monte Carlo probabilistic sampling in its ML pipeline. This approach improved the production of linalool and geraniol in *S. cerevisiae*, enabling the engineering of strains capable of producing hoppy beer through heterologous expression (Radivojević et al. 2020). In another application, ART and the EVOLVE algorithm were combined with a genome-scale model to improve tryptophan production. Using a combinatorial library of six promoters and five genes, these ML models achieved 74% and 43% improvements in tryptophan titer and productivity, respectively (Zhang et al. 2020). Similarly, genome-scale modeling and ML (GSM-ML) were applied to *Y. lipolytica* using publicly available genetic engineering and production data. The model showed high predictive accuracy and demonstrated potential for extension to other non-model oleaginous yeasts (Czajka et al. 2021).

In yeast terpenoid production, a RF algorithm was used to identify optimal promoter combinations for overexpressing non-rate-limiting enzymes in the MVA pathway. RF analysis pinpointed three non-rate-limiting enzymes influencing geraniol production and predicted promoter combinations that significantly increased higher geraniol levels. This combinatorial and ML platform was subsequently applied to other terpenoids, including sesquiterpene, α-humulene, triterpene, and squalene (Mukherjee et al. 2022). A more complex stacking ML approach, combining 24 algorithms across three metamodel layers, was implemented to optimize carotenoid bioproduction, achieving higher titers than rule-based tuning in S. *cerevisiae*. The model accurately predicted carotenoid productivity based on gene expression profiles from the combinatorial promoter library, achieving up to a 4.3-fold increase in yield. It also identified genes with the greatest influence on production (Shimazaki et al. 2024). A similar stacking ML strategy was successfully applied to optimize D-lactic acid production, using gene expression data for D-lactate dehydrogenase and glycolytic enzymes as model inputs (Yamamoto et al. 2023).

Recently, the gradient boosting algorithm, XGBoost, was applied to optimize bioethanol production in S. *cerevisiae*. Fine-tuning the native promoter of pyruvate carboxylase and alcohol dehydrogenase genes, key rate-limiting genes, led to a 63% increase in ethanol yield. The ML workflow also reduced experimental workload, narrowing down the number of strains tested under heat stress (40°C) during a second DBTL cycle (Khamwachirapithak et al. 2023). In another study, heterologous genes from *E. coli* and *Flavobacterium johnsoniae* and plant *Arabidopsis thaliana* were co-optimized in *S. cerevisiae* using various regression models, including multiple linear regression, support vector regression, kernel ridge regression, and RF regression, to enhance *p*-coumaric acid production. By screening only 13.5% of a combinatorial library, the training models effectively predicted high-performing gene expression combinations (Moreno-Paz et al. 2024).

In microbial cell factory engineering, ML enables the discovery of complex relationships in the pathway design, strain optimization, and bioprocess development. Integrating ML across all stages of the DBTL cycle reduces development time, improves design success rates, and broadens the range of engineerable yeasts and target compounds. As more annotated datasets, particularly from non-model species, become available, the predictive accuracy and generalizability of ML models will continue to improve. While ML can uncover hidden patterns without prior knowledge of metabolic pathways or biochemical mechanisms, its performance depends heavily on data quality and quantity. Poor training data can lead to underfitting or overfitting, reducing predictive power. Additionally, deep learning models such as neural networks, though powerful for complex optimization tasks, are less interpretable than traditional algorithms like multiple linear regression, SVM, RF, or XGBoost. Furthermore, deep learning requires a significantly higher volume of data compared to traditional ML, as its objective is to resolve greater pattern complexity. Consequently, constructing large deep learning models requires powerful computational resources and extended training periods. Limited access to such computing infrastructure can directly hinder subsequent experimental validation

## Integrating ML and biodiversity: a data-driven approach to advance yeast engineering

Traditional metabolic engineering in both model and non-model yeasts often relies on empirical, trial-and-error DBTL cycles, which are slow, labor-intensive, and poorly suited for complex pathways (Table 1). A major challenge in metabolic engineering is achieving dynamic pathway control. Static overexpression often disrupts cellular balance, leading to cofactor imbalances, toxic intermediates, and reduced yields. While systems like dynamic sensor-regulator systems (DSRS) have worked in bacteria, their application in yeast is limited by the lack of modular, tunable components compatible with eukaryotic transcription. Bridging this gap requires a broader set of inducible promoters, RNA-based regulators, and context-specific elements responsive to intracellular signals (Sheng and Feng 2015).

ML, combined with expanding genomic and phenotypic data from diverse yeasts, offers a powerful solution to current metabolic engineering challenges (Table 2). ML can identify patterns in complex datasets to predict system behavior and guide the rational design of genetic circuits, metabolic pathways, and fermentation strategies. Integration with omics data also allows system-level modeling of metabolism and stress responses, revealing new engineering opportunities and potential host strains. The integration of ML into yeast metabolic engineering marks a shift from empirical methods to data-driven strategies. As tools and datasets advance, the DBTL cycle becomes faster, more predictive, and scalable, accelerating the creation of next-generation microbial cell factories. This section highlights how ML aids in discovering and engineering genetic parts and chassis from yeast biodiversity (see subsection “ML-guided discovery of genetic parts from yeast biodiversity”), and how it drives yeast engineering workflows throughout the DBTL process (see subsection “ML applications across yeast engineering workflows”).

**Table 2.** Comparison of the limitations of traditional metabolic engineering and ML-driven solutions.

Aspect	Traditional metabolic engineering	ML-driven solutions
Design approach	Empirical, intuition-based	Predictive, data-driven
Efficiency	Slow, labor-intensive DBTL	Accelerated via prediction and prioritization
Pathway balancing	Static control often causes imbalances	Supports dynamic, optimized regulation (e.g. DSRS)
Regulatory elements	Limited, hard to tune	Enables design of synthetic, tunable, context-specific parts
Strain optimization	Gene/pathway changes made one at a time	Multi-gene optimization via combinatorial approaches
Use of omics data	Rarely used due to complexity	Integrates omics for system-level insight
Adaptability to non-model yeasts	Hindered by limited tools	Enables de novo designs with minimal data
Prediction accuracy	Low; depends on repeated trials	High; trained on experimental datasets
Scalability	Scalable through automation	Scalable via automation and algorithms
Strain stability	Often overlooked	Predicts long-term behavior and stability

### ML-guided discovery of genetic parts from yeast biodiversity

Yeast species exhibit vast natural diversity, spanning thousands of Ascomycota and Basidiomycota members adapted to varied niches such as fermented foods, insect guts, and marine environments. This diversity results in broad physiological and metabolic traits. Non-conventional yeasts like *Y. lipolytica, Scheffersomyces stipitis, K. marxianus, P. pastoris*, and *Lipomyces starkeyi* offer valuable characteristics, including thermotolerance, lipid accumulation, and alternative carbon utilization (Bankar et al. 2009, Mukherjee et al. 2017, Cernak et al. 2018, Sun et al. 2020, de Oliveira et al. 2021). Mining this diversity offers a largely untapped reservoir of biosynthetic enzymes, regulatory elements, transporters, and stress-tolerance mechanisms that can expand the current genetic toolkit for strain engineering (Hubmann et al. 2013, Roullier-Gall et al. 2020, Lu et al. 2021, Segal-Kischinevzky et al. 2022, Siddiq and Wittkopp 2022, Al Halim et al. 2024). Despite this potential, the practical exploitation of yeast biodiversity has been constrained by a lack of scalable, high-throughput methods to identify and validate functionally relevant genetic elements.

ML, particularly deep learning and ensemble methods like RFs and gradient boosting, now offers a transformative solution by enabling high-throughput prediction and prioritization of functional parts from genomic and phenotypic data. By learning patterns from characterized sequence-function relationships, ML can infer the likely function of unknown elements, accelerate regulatory motif annotation, and even design synthetic elements with tailored properties. One promising application of ML is identifying and characterizing native regulatory parts such as promoters, terminators, and untranslated regions (UTRs) from underexplored yeast genomes (Table 3). For instance, transcriptomic studies have uncovered inducible promoters in *X. dendrorhous* for carotenoid biosynthesis under oxidative stress conditions (Tobin et al. 2024). In *O. polymorpha*, mined promoter–terminator combinations enable stable and tunable expression under methanol or glucose, supporting its development as a methylotrophic chassis (Wefelmeier et al. 2022). Although initially based on transcriptomic data, ML can expand these efforts by predicting regulatory motifs across species, enabling direct design of functional parts in non-model yeasts.

**Table 3.** Examples of ML used in discovery and identification of genetic parts.

Area of Application	Research Purposes	ML methods	Organism*	References
Genetic parts	Differentiating promoter from non-promoter	Random forest, ResNet (Deep learning)	S. *cerevisiae*, *A. thaliana*, and *Homo sapiens*	Bhandari et al. (2021), Liu et al. (2022)
	Predicting promoter strengths or transcript levels	CNN	*S. cerevisiae* and *P. pastoris*	de Boer et al. (2020), Kotopka and Smolke (2020), Zrimec et al. (2022), Cheng et al. (2025)
	Predicting essential gene	Random forest Gradient boosting, Support vector machine	*S. cerevisiae*, *S. pombe*, and *C. albicans*	Campos et al. (2019), Levitan et al. (2020)
	Predicting protein production	XGBoost, Multi-layer perceptron elastic net regression	*K. marxianus*, *S. pombe*	Ferreira et al. (2021), May et al. (2023), Zeng et al. (2024)
	Identifying of gene function	ExtraTreesRegression, Tree-based regression	*S. pombe*	Dickinson et al. (2022), Rodríguez-López et al. (2023)
Gene regulation	Predicting DNA binding affinity	Random forest regressor	*S. cerevisiae*	Barissi et al. (2022)
	Predicting transactivation domain	CNN	*S. cerevisiae*	Erijman et al. (2020)

* Listed organisms represent those in which ML or synthetic biology methods were developed or validated; approaches are generally transferable to other yeasts.

Deep learning models trained on large sequence-function datasets from *S. cerevisiae* have shown impressive generalizability. For example, a ML pipeline using deep learning and RFs with one-hot encoding accurately distinguished promoter from non-promoter sequences across genomes of *S. cerevisiae, A. thaliana*, and *Homo sapiens* (Bhandari et al. 2021). To address deep learning’s reliance on large training datasets, transfer learning and deep residual networks (ResNet) were applied for promoter prediction, offering an alternative ML pipeline for yeast promoter identification (Liu et al. 2022). To fine-tune yeast expression systems, ML models trained on high-throughput datasets have been used to predict promoter strength. Convolutional neural networks (CNNs) trained on >100 million synthetic constitutive and inducible promoters achieved high predictive accuracy (*R*² > 0.79) (de Boer et al. 2020). Similarly, generative models have designed novel promoters with greater dynamic ranges than their native counterparts (Zrimec et al. 2022). Although developed for *S. cerevisiae*, these models can be adapted to other yeasts using transfer learning, enabling direct design in non-model hosts and avoiding cross-species limitations. For example, in *P. pastoris*, a CNN trained on the alcohol oxidase 1 promoter identified key motifs that led to a mutant library with a 5.3-fold strength increase over the wild type (Cheng et al. 2025).

In parallel, ML has also been effective in characterizing promoter strength. A CNN trained on synthetic promoter libraries, varying TF binding sites, spacer lengths, and TATA motifs, accurately predicted promoter activity and identified key motifs (Kotopka and Smolke 2020). In *E. coli*, a synthetic promoter library derived from Trc promoters demonstrated a 454-fold activity range across 3665 promoter variants, with strengths accurately predicted using XGBoost (Zhao et al. 2022). These studies highlight the potential of ML, especially deep learning and gradient boosting, for accurate promoter prediction based on diverse sequence features.

Beyond regulatory elements, ML enables the identification of essential genes and proteins. RF classifiers trained on transposon mutagenesis data from *S. cerevisiae, S. pombe*, and *C. albicans* successfully predicted gene essentiality (Levitan et al. 2020, Beder et al. 2021). These models, validated even in non-yeast species, demonstrate strong generalizability. Protein abundance profiles from *S. cerevisiae* have also been used to train models extended to *K. marxianus* and *S. pombe*, with high correlation and robustness (Ferreira et al. 2021). ML facilitates gene function prediction by integrating multi-omics data. Combining proteomic and metabolomic profiles with tree-based regression models effectively predicted novel gene functions in yeast strains (Dickinson et al. 2022). It also uncovers new biosynthetic genes and pathways in non-conventional yeasts. For example, ML classifiers applied genomic and phenotypic data from 182 yeast species identified uncharacterized xylose transporters (Fiamenghi et al. 2022). Similarly, genotype-phenotype analysis of >1000 genomes revealed a non-canonical galactose utilization pathway in *Ogataea* and *Candida* species, independent of the classical GAL network (Harrison et al. 2024).

ML enhances understanding of gene regulation in yeasts. For instance, ML models trained on expression data from synthetic yeast promoters in *S. cerevisiae* accurately predicted regulatory logic involving TFs (de Boer et al. 2020). RF models using *in vitro* TF binding assay data predicted TF-DNA binding affinities (Barissi et al. 2022). Deep learning also identified acidic transcription activation domains from peptide sequences (Erijman et al. 2020), and predicted protein production from 5′ UTR poly(A) sequences in *K. marxianus* (Zeng et al. 2024). Beyond parts and pathways, ML offers a systematic approach for host strain selection. Traditional criteria such as GRAS status, transformation efficiency, and literature familiarity often bias strain choice toward a few well-studied species. ML enables more objective predictions of strain suitability by modeling traits such as metabolic capabilities, stress tolerance, and compatibility with synthetic biology tools. Multi-omics datasets from native isolates train classifiers to evaluate biotechnological potential, even before strain domestication.

Integrating ML with yeast biodiversity creates a powerful closed-loop framework that transforms untapped genetic diversity into a practical resource for building robust microbial cell factories. As more yeast strains are sequenced and characterized, ML can efficiently prioritize the selection of genes, regulatory parts, metabolic pathways, and optimal host organisms. For example, promoters and terminators isolated from extremophilic yeasts can yield regulatory elements that withstand industrial stresses, such as acidic conditions and high temperatures. Additionally, orthogonal circuits inspired by diverse yeast networks support modular, multi-layered pathway designs that are essential for sophisticated metabolic engineering tasks. This combined approach not only accelerates the discovery of novel genetic components but also fundamentally reshapes the way microbial hosts are selected, engineered, and optimized. As a result, it helps overcome the current bottleneck in strain development, enabling the full potential of non-conventional yeasts to be harnessed for sustainable biomanufacturing.

### ML applications across yeast engineering workflows

After selecting genetic parts and chassis strains, the next challenge involves engineering metabolic pathways. This task remains complex even in well-characterized organisms like *S. cerevisiae*, as optimizing multiple genes simultaneously for improved yield, stability, and robustness is difficult. ML is improving every step in the DBTL cycle through predictive modeling, informed decision-making based on data, and continuous refinement. During the Design stage, ML models predict how genetic constructs might behave even before they are synthesized. They assist in choosing optimal promoters and ribosome binding sites, estimating mRNA stability, and optimizing codon usage to improve protein expression (Zrimec et al. 2022, Yan et al. 2024). In pathway engineering, ML also models enzyme expression interactions to balance metabolic flux and improve pathway performance. The Build and Test stages produce essential experimental data, including gene expression levels, product titers, and growth rates. These data are then analyzed in the Learn stage using supervised ML algorithms, such as RFs, and XGBoost and deep learning. These models identify patterns linking genetic features to phenotypic outcomes, guiding iterative design improvements and accelerating strain optimization.

Predicting yeast phenotypes from genomic data enables more effective chassis selection and leverages distinctive growth characteristics available in yeast biodiversity. Grinberg and team applied five ML algorithms to predict yeast growth across 46 conditions, outperforming traditional statistical genetics (Grinberg et al. 2020). Another research using the identical yeast dataset has identified positive- and negative-growth associated markers by relevance vector machine ensemble (Ayat and Domaratzki 2022). ML combined with multi-omics data has demonstrated strong potential in phenotype prediction, especially growth rate. A large-scale multiomics study integrated metabolic modeling with transcriptomic and metabolomic data from 1143 *S. cerevisiae* mutants (Culley et al. 2020). Using algorithms such as SVMs, RFs, and neural networks, the study successfully predicted growth across diverse yeast collections with similar omics profiles. Similarly, transcriptional profiles have been used to predict growth rates in *E. coli* and *S. cerevisiae* via k-nearest neighbors regression (Wytock and Motter 2019). These approaches facilitate prediction of growth and metabolic traits in diverse yeasts from gene expression or omics signatures. Beyond growth, ML with omics data has predicted other phenotypes; for example, in *Candida auris*, ML on whole-genome sequences of 358 strains identified drug resistance mutations and novel variants (Li et al. 2021a). Similarly, XGBoost analysis of genomic data from 182 Saccharomycotina strains identified four new xylose transporters, which are potentially valuable for lignocellulosic biomass utilization (Fiamenghi et al. 2022).

Phenomic data, when combined with ML methods, have also proven effective in predicting gene function in *S. pombe*. The integrated ML pipeline (NET-FF) combined sequence homology data with phenotypic outcomes from non-essential gene deletions to predict Gene Ontology terms. This approach successfully identified novel proteins whose functions were confirmed experimentally (Rodríguez-López et al. 2023). ML also predicts context-dependent responses and uncovers regulatory circuits tied to environmental resilience. For instance, models trained on stress-induced transcriptomic data have identified TFs and gene modules associated with tolerance to stresses like high temperature or low pH tolerance in species like *K. marxianus* and *I. orientalis* (Dekker et al. 2021, Tan et al. 2025). Overall, integrating ML with multi-omics data allows accurate phenotype prediction, facilitates the discovery of new metabolic pathways, streamlines strain selection, and aids in identifying novel yeast chassis.

Beyond biological data, ANNs have demonstrated capabilities in image recognition and classification (Rawat and Wang 2017). This functionality has been applied in various studies. A gradient boosting decision Tree (GBDTs) and ANNs were used to predict bioethanol production from morphological images. While both models achieved comparable real-time prediction accuracy, the ANN outperformed GBDTs in forecasting bioethanol levels at 30 and 60 min after image acquisition (Itto-Nakama et al. 2022). CNNs have recently been employed to classify seven different yeast species, including *C. albicans, S. cerevisiae, Geotrichum candidum, Rhodotorula babjevae, Y. lipolytica, Debaryomyces hansenii*, and *Wickerhamomyces anomalus*, using microcolony images. This image-based method provides a fast and accurate alternative to conventional molecular identification techniques (Park et al. 2025). *Candida* species were also identified using single-cell microscopic images and CNN method (Shankarnarayan and Charlebois 2024). In addition to optimizing pathways and strains, ML has emerged as a valuable tool beyond traditional synthetic biology applications, notably in yeast identification and taxonomy through image-based deep learning.

As ML tools become more accessible and datasets from non-model yeast species grow, incorporating these resources into strain design workflows is significantly transforming metabolic engineering approaches (Table 4). The ML pipeline integrates biodiversity datasets to uncover novel biological functions and guide metabolic engineering strategies (Fig. 3). It involves five key steps: data collection, data preprocessing, feature extraction, model training and validation, and application in metabolic engineering, where model-derived insights are applied to strain design and pathway optimization. The combined impact includes reduced development time, higher design success rates, and broader applicability to diverse yeasts and complex products. The integration of ML with yeast genomic diversity represents a major shift toward predictive, high-throughput strain design informed by naturally evolved genetic traits. Utilizing data-driven tools and multi-omics insights enables researchers to explore beyond conventional yeast models, accessing a broader array of microbial hosts, metabolic pathways, and novel phenotypes. Furthermore, ML is now frequently employed in bioprocess optimization tasks. These include modeling fermentation kinetics, predicting yields under different pH and oxygen regimes, and controlling real-time feeding strategies. With improvements in sensor technology and automation, ML-driven process control is becoming increasingly common and is expected to become a standard feature in precision fermentation workflows (Helleckes et al. 2023, Chai et al. 2024). By combining data-driven tools with naturally evolved traits, researchers can overcome the limitations associated with model organisms. This integration expands the range of host organisms, genetic parts, and design strategies available for engineering. As annotated datasets grow and ML models become more widely applicable across yeast species, this combined approach is expected to create new opportunities for developing scalable, sustainable, and robust microbial platforms.

**Table 4.** Examples of ML used in pathway design and phenotype prediction.

Area of Application	Research Purposes	ML methods	Organism*	References
Phenotype prediction	Predicting yeast growth	Support vector machine, Random forest, Artificial neural network, relevance vector machine ensemble	*S. cerevisiae*, Saccharomycotina subphylum	Culley et al. (2020), Grinberg et al. (2020), Ayat and Domaratzki (2022), Harrison et al. (2024)
	Predicting drug resistance from genomic sequences	ML algorithms	*C. auris* and Saccharomycotina subphylum	Fiamenghi et al. (2022)
Metabolic engineering	Optimizing expression fine-tuning for enhanced bioproduction.	ART, Random Forest, XGBoost, multiple linear regression, and support vector regression	*S. cerevisiae* and *Y. lipolytica*	Radivojević et al. (2020), Zhang et al. (2020), Czajka et al. (2021), Mukherjee et al. (2022), Khamwachirapithak et al. (2023), Moreno-Paz et al. (2024), Shimazaki et al. (2024)
Image analysis	Predicting of ethanol fermentation from morphological image	Gradient boosting Decision Tree, Artificial neural network	*S. cerevisiae*	Itto-Nakama et al. (2022)
	Segmentation	CNN	*S. cerevisiae*	Dietler et al. (2020), Bunk et al. (2022)
	Identifying yeast strains/species identification	CNN	*C. albicans*, *S. cerevisiae*, *G. candidum*, *R. babjevae*, *Y. lipolytica*, *D. hansenii*, and *W. anomalus*	Shankarnarayan and Charlebois (2024), Park et al. (2025)

* Listed organisms represent those in which ML or synthetic biology methods were developed or validated; approaches are generally transferable to other yeasts.

## Future directions and perspectives

Looking ahead, the integration of ML with the broad genetic and metabolic diversity of yeasts is likely to reshape the landscape of yeast synthetic biology. To realize this potential, further advances in ML models are essential. A key priority is the development of models capable of integrating multi-omics data—genomic, transcriptomic, proteomic, and metabolomic—to provide more comprehensive predictive power. Previous studies have shown that such integration can improve pathway prediction accuracy and even outperform traditional kinetic models. Soon, we expect to see “mechanism-aware” ML models that incorporate biological knowledge, such as known metabolic pathways or regulatory networks, into the learning process. These hybrid approaches combine data-driven learning with system-level constraints, enabling more accurate and interpretable predictions (Culley et al. 2020).

Equally important is the application of generative algorithms for the design of biological parts. Rather than only analyzing existing sequences, ML models—particularly deep generative networks—will be used to generate new regulatory elements or even synthetic pathways (Zrimec et al. 2022). These early examples suggest that future ML-guided design tools may soon come up with novel sequences and protein variants that go beyond conventional engineering methods. Another important area will be cross-species generalization. ML models will need to learn principles from one or several well-characterized yeasts and apply them to predict behaviors in lesser-known species. With the right training on large and diverse datasets, models may move beyond *S. cerevisiae*-focused applications to enable rational engineering in non-model yeasts—without the need to restart tool development for each new host. Achieving this goal will require progress in feature learning, and cross-species benchmarking.

By leveraging yeast biodiversity, ML opens several promising areas of research. One major opportunity is genome mining for new metabolic functions. ML combined with comparative genomics has already helped discover unknown genes in various yeast clades. This kind of approach could be extended to many other functions, including metabolite transport and stress response. Another key direction is the discovery and design of regulatory parts. Because eukaryotic regulatory sequences are structurally complex, their optimization has been limited by conventional methods. ML models trained on large sequence-function datasets can map out the hidden “rules” of regulatory DNA and assist in designing stronger, more tunable promoters and terminators. Generative approaches could be used to suggest regulatory elements that are tailored for specific yeasts or designed to respond to environmental cues.

In addition, the ability to predict gene function in different contexts represents another valuable application. Many genetic features—such as enzyme efficiency or pathway relevance—are influenced by environmental or organism-specific factors. ML models that incorporate phylogenetic or environmental information are starting to show potential in predicting how a gene or enzyme will behave under various conditions. For example, ML has been used to estimate enzyme kinetics (*k*_cat_ values) based on protein sequences (Li et al. 2022b). These tools could soon allow researchers to simulate how a modification in one yeast might behave in another or predict how a metabolic network will adapt under stress, providing valuable insights for strain engineering.

We contend that one of the most transformative impacts of ML may be its ability to extend synthetic biology beyond the limited group of model yeast species currently in use. Currently, engineering efforts focus mainly on a few species, but there are more than a thousand known yeast species, many of which offer unique phenotypes and biotechnological potential. With the help of biodiversity-aware datasets, ML tools could identify new parts, pathways, and host candidates with minimal experimental screening. Such discoveries allow researchers to choose hosts with the traits they need, rather than engineering them from scratch.

A logical next step is expanding the use of synthetic biology tools to methylotrophic and extremophilic yeasts. *O. polymorpha*, for instance, can use methanol as a sole carbon and energy source, and with methanol production from CO₂ and hydrogen becoming more feasible, this yeast has received growing attention for sustainable bioprocessing (Xie et al. 2024). ML could be used to rapidly identify promoters, pathways, and adaptive traits in *O. polymorpha* and other non-conventional yeasts for carbon-negative applications. Moreover, ML-guided genomic comparisons can help identify yeasts that are naturally resistant to stressors like high temperatures, salt, or fermentation inhibitors—traits that are useful for lowering production costs and improving process robustness.

In parallel with these modeling advances, we also expect the experimental workflows to change. Closed-loop, automated DBTL cycles are becoming increasingly standard in synthetic biology (Spannenkrebs et al. 2025). In these systems, ML serves as the “brain” of the operation, generating genetic designs based on existing data. High-throughput automation then performs the construction and testing of these designs. This setup can accelerate strain engineering by reducing the design space and making the experiments more focused. Although the number of possible pathway combinations can be vast, ML methods can prioritize only the most promising variants for testing. One challenge, however, has been the mismatch in speed between design recommendations and experimental validation. Closed-loop platforms solve this by linking real-time decision-making with automated implementation. As these platforms continue to evolve, we can expect continuous DBTL cycles in which ML models update in real-time based on new data. This will not only accelerate strain optimization but also enhance model performance with each iteration.

The convergence of ML and synthetic biology will enable several impactful applications. One is the production of complex natural products, such as terpenoids and alkaloids, which often involve multi-gene pathways. ML can aid in pathway balancing, enzyme selection, and even protein engineering, enabling yeast to synthesize these compounds more efficiently. Another opportunity is carbon-negative fermentation. Future strains may be engineered to use C1 feedstocks like methanol, formate, or CO_2_. ML will be central to identifying and tuning the genetic components that enable these transformations. A third major frontier is the creation of adaptive cell factories. Industrial bioprocesses often expose cells to variable temperatures, contaminants, or fluctuating feedstocks. With sufficient training data from stress-tolerant yeasts, ML models could guide the design of genetic circuits that respond dynamically to changing environments. This would support strain deployment in low-cost, variable settings, where robustness is as critical as productivity.

Taken together, the intersection of ML and yeast biodiversity represents a major shift in how synthetic biology will be done. By expanding the design space with nature’s diversity and accelerating the DBTL cycle with smart algorithms, future engineering efforts can become more predictable, efficient, and scalable. In the coming years, we anticipate that yeast synthetic biology will shift from largely manual and trial-based workflows to a more automated, data-driven discipline. With the help of ML, the field can explore bolder ideas, reduce time to implementation, and unlock the full potential of yeast as a platform for bioproduction.

## Data Availability

No new data were generated or analysed in support of this research.

## References

[bib1] Al Halim LRA, Hemeda NF, Serag AM. Isolation, characterization, and screening of yeast biodiversity for multi- hydrolytic enzymes. JUmm Al-Qura Univ Appll Sci. 2024;10:474–84. 10.1007/s43994-023-00118-6.

[bib2] Aravind K, Naik TP, Krishnaswamy K. Exopolysaccharide production potential of yeast *Cryptococcus flavescens* SK01 strain from the phylloplane of *Semecarpus kathalekanensis*. J Pure Appl Microbiol. 2014;8:3339–43.

[bib3] Ayat M, Domaratzki M. Sparse bayesian learning for genomic selection in yeast. Front Bioinform. 2022;2:960889. 10.3389/fbinf.2022.960889. 36304259 PMC9580947

[bib4] Bankar AV, Kumar AR, Zinjarde SS. Environmental and industrial applications of *Yarrowia lipolytica*. Appl Microbiol Biotechnol. 2009;84:847–65. 10.1007/s00253-009-2156-8. 19669134

[bib5] Barissi S, Sala A, Wieczór M et al. DNAffinity: a machine-learning approach to predict DNA binding affinities of transcription factors. Nucleic Acids Res. 2022;50:9105–14. 10.1093/nar/gkac708. 36018808 PMC9458447

[bib6] Beder T, Aromolaran O, Dönitz J et al. Identifying essential genes across eukaryotes by machine learning. NAR Genom Bioinform. 2021;3:lqab110. 10.1093/nargab/lqab110. 34859210 PMC8634067

[bib7] Benmessaoud S, Anissi J, Kara M et al. Isolation and characterization of three new crude oil degrading yeast strains, *Candida parapsilosis* SK1, *Rhodotorula mucilaginosa* SK2 and SK3. Sustainability. 2022;14:3465. 10.3390/su14063465.

[bib8] Bernauer L, Radkohl A, Lehmayer LGK et al. *Komagataella phaffii* as emerging model organism in fundamental research. Front Microbiol. 2021;11:607028. 10.3389/fmicb.2020.607028. 33505376 PMC7829337

[bib9] Bhandari N, Khare S, Walambe R et al. Comparison of machine learning and deep learning techniques in promoter prediction across diverse species. PeerJ Comput Sci. 2021;7:e365. 10.7717/peerj-cs.365. PMC7959599 33817015

[bib10] Boekhout T, Amend AS, El Baidouri F et al. Trends in yeast diversity discovery. Fungal Divers. 2022;114:491–537. 10.1007/s13225-021-00494-6.

[bib11] Bratiichuk D, Kurylenko O, Vasylyshyn R et al. Development of new dominant selectable markers for the nonconventional yeasts *Ogataea polymorpha* and *Candida famata*. Yeast. 2020;37:505–13. 10.1002/yea.3467. 32307750

[bib12] Bunk D, Moriasy J, Thoma F et al. YeastMate: neural network-assisted segmentation of mating and budding events in *Saccharomyces cerevisiae*. Bioinformatics. 2022;38:2667–9. 10.1093/bioinformatics/btac107. 35179572 PMC9048668

[bib13] Campos TL, Korhonen PK, Gasser RB et al. An evaluation of machine learning approaches for the prediction of essential genes in eukaryotes using protein sequence-derived features. Comput Struct Biotechnol J. 2019;17:785–96. 10.1016/j.csbj.2019.05.008. 31312416 PMC6607062

[bib14] Cao L, Li J, Yang Z et al. A review of synthetic biology tools in *Yarrowia lipolytica*. World J Microbiol Biotechnol. 2023;39:129. 10.1007/s11274-023-03557-9. 36944859

[bib15] Cernak P, Estrela R, Poddar S et al. Engineering *Kluyveromyces marxianus* as a robust synthetic biology platform host. mBio. 2018;9:e01410–18. 10.1128/mbio.01410-18. 30254120 PMC6156195

[bib16] Chai WY, Tan MK, Teo KTK et al. Optimization of fed-batch baker’s yeast fermentation using deep reinforcement learning. Process Integr Optim Sustain. 2024;8:395–411. 10.1007/s41660-024-00406-6.

[bib17] Chen Q, Lyu L, Xue H et al. Engineering a non-model yeast *Rhodotorula mucilaginosa* for terpenoids synthesis. Synthetic Syst Biotechnol. 2024;9:569–76. 10.1016/j.synbio.2024.04.015. PMC11058065 38690180

[bib18] Chen Y, Banerjee D, Mukhopadhyay A et al. Systems and synthetic biology tools for advanced bioproduction hosts. Curr Opin Biotechnol. 2020;64:101–9. 10.1016/j.copbio.2019.12.007. 31927061

[bib19] Cheng Z, Wu W, Zhou X et al. Microfluidic fluorescence-activated cell sorting of convolutional neural network-designed synthetic yeast promoter. ACS Synth Biol. 2025;14:2105–16. 10.1021/acssynbio.5c00025. 40368332

[bib20] Cui Z, Zheng H, Zhang J et al. A CRISPR/Cas9-mediated, homology-independent tool developed for targeted genome integration in *Yarrowia lipolytica*. Appl Environ Microb. 2021;87:e02666–20. 10.1128/AEM.02666-20. PMC8105018 33452022

[bib21] Culley C, Vijayakumar S, Zampieri G et al. A mechanism-aware and multiomic machine-learning pipeline characterizes yeast cell growth. Proc Natl Acad Sci USA. 2020;117:18869–79. 10.1073/pnas.2002959117. 32675233 PMC7414140

[bib22] Czajka JJ, Oyetunde T, Tang YJ. Integrated knowledge mining, genome-scale modeling, and machine learning for predicting *Yarrowia lipolytica* bioproduction. Metab Eng. 2021;67:227–36. 10.1016/j.ymben.2021.07.003. 34242777

[bib23] de Boer CG, Vaishnav ED, Sadeh R et al. Deciphering eukaryotic gene-regulatory logic with 100 million random promoters. Nat Biotechnol. 2020;38:56–65. 10.1038/s41587-019-0315-8. 31792407 PMC6954276

[bib24] Decoene T, Brecht DP, Jo M et al. Standardization in synthetic biology: an engineering discipline coming of age. Crit Rev Biotechnol. 2018;38:647–56. 10.1080/07388551.2017.1380600. 28954542

[bib25] Dekker WJC, Ortiz-Merino RA, Kaljouw A et al. Engineering the thermotolerant industrial yeast *Kluyveromyces marxianus* for anaerobic growth. Metab Eng. 2021;67:347–64. 10.1016/j.ymben.2021.07.006. 34303845

[bib26] de Oliveira PM, Aborneva D, Bonturi N et al. Screening and growth characterization of non-conventional yeasts in a hemicellulosic hydrolysate. Front Bioeng Biotechnol. 2021;9:659472. 10.3389/fbioe.2021.659472. 33996782 PMC8116571

[bib27] Dickinson Q, Kohler A, Ott M et al. Multi-omic integration by machine learning (MIMaL). Bioinformatics. 2022;38:4908–18. 10.1093/bioinformatics/btac631. 36106996 PMC9801967

[bib28] Dietler N, Minder M, Gligorovski V et al. A convolutional neural network segments yeast microscopy images with high accuracy. Nat Commun. 2020;11:5723. 10.1038/s41467-020-19557-4. 33184262 PMC7665014

[bib29] Dixon TA, Pretorius IS. Drawing on the past to shape the future of synthetic yeast research. Int J Mol Sci. 2020;21:7156. 10.3390/ijms21197156. 32998303 PMC7583028

[bib30] Dou W, Zhu Q, Zhang M et al. Screening and evaluation of the strong endogenous promoters in *Pichia pastoris*. Microb Cell Fact. 2021;20:156. 10.1186/s12934-021-01648-6. 34372831 PMC8351359

[bib31] Erijman A, Kozlowski L, Sohrabi-Jahromi S et al. A high-throughput screen for transcription activation domains reveals their sequence features and permits prediction by deep learning. Mol Cell. 2020;78:890–902.e6. e6. 10.1016/j.molcel.2020.04.020. 32416068 PMC7275923

[bib32] Eskandari A, Nezhad NG, Leow TC et al. Current achievements, strategies, obstacles, and overcoming the challenges of the protein engineering in *Pichia pastoris* expression system. World J Microbiol Biotechnol. 2024;40:39. 10.1007/s11274-023-03851-6. 38062216

[bib33] Ferreira M, Ventorim R, Almeida E et al. Protein abundance prediction through machine learning methods. J Mol Biol. 2021;433:167267. 10.1016/j.jmb.2021.167267. 34563548

[bib34] Fiamenghi MB, Bueno JGR, Camargo AP et al. Machine learning and comparative genomics approaches for the discovery of xylose transporters in yeast. Biotechnol Biofuels. 2022;15:57. 10.1186/s13068-022-02153-7. PMC9123741 35596177

[bib35] Gao J, Gao N, Zhai X et al. Recombination machinery engineering for precise genome editing in methylotrophic yeast *Ogataea polymorpha*. iScience. 2021;24:102168. 10.1016/j.isci.2021.102168. 33665582 PMC7907465

[bib36] Gao J, Li Y, Yu W et al. Rescuing yeast from cell death enables overproduction of fatty acids from sole methanol. Nat Metab. 2022;4:932–43. 10.1038/s42255-022-00601-0. 35817856

[bib37] Greener JG, Kandathil SM, Moffat L et al. A guide to machine learning for biologists. Nat Rev Mol Cell Biol. 2022;23:40–55. 10.1038/s41580-021-00407-0. 34518686

[bib38] Grinberg NF, Orhobor OI, King RD. An evaluation of machine-learning for predicting phenotype: studies in yeast, rice, and wheat. Mach Learn. 2020;109:251–77. 10.1007/s10994-019-05848-5. 32174648 PMC7048706

[bib39] Grondin E, Shum C, Sing A et al. A comparative study on the potential of epiphytic yeasts isolated from tropical fruits to produce flavoring compounds. Int J Food Microbiol. 2015;203:101–8. 10.1016/j.ijfoodmicro.2015.02.032. 25802220

[bib40] Guardiola FA, MÁ E, Angulo C. *Yarrowia lipolytica*, health benefits for animals. Appl Microbiol Biotechnol. 2021;105:7577–92. 10.1007/s00253-021-11584-5. 34536101

[bib41] Harrison M-C, Ubbelohde EJ, LaBella AL et al. Machine learning enables identification of an alternative yeast galactose utilization pathway. Proc Natl Acad Sci USA. 2024;121:e2315314121. 10.1073/pnas.2315314121. 38669185 PMC11067038

[bib42] Helleckes LM, Hemmerich J, Wiechert W et al. Machine learning in bioprocess development: from promise to practice. Trends Biotechnol. 2023;41:817–35. 10.1016/j.tibtech.2022.10.010. 36456404

[bib43] Hou S, Yang S, Bai W. Multi-gene precision editing tool using CRISPR-Cas12a/Cpf1 system in *Ogataea polymorpha*. Microb Cell Fact. 2025;24:28. 10.1186/s12934-025-02654-8. 39838422 PMC11748851

[bib44] Hubmann G, Foulquié-Moreno MR, Nevoigt E et al. Quantitative trait analysis of yeast biodiversity yields novel gene tools for metabolic engineering. Metab Eng. 2013;17:68–81. 10.1016/j.ymben.2013.02.006. 23518242

[bib45] Itto-Nakama K, Watanabe S, Kondo N et al. AI-based forecasting of ethanol fermentation using yeast morphological data. Biosci Biotechnol Biochem. 2021;86:125–34. 10.1093/bbb/zbab188. 34751736

[bib46] Jach ME, Malm A. *Yarrowia lipolytica* as an alternative and valuable source of nutritional and bioactive compounds for humans. Molecules. 2022;27:2300. 10.3390/molecules27072300. 35408699 PMC9000428

[bib47] Jaiboon K, Lertwattanasakul N, Limtong P et al. Yeasts from peat in a tropical peat swamp forest in Thailand and their ability to produce ethanol, indole-3-acetic acid and extracellular enzymes. Mycol Progress. 2016;15:755–70. 10.1007/s11557-016-1205-9.

[bib48] Jia N, Ni X, Wang H et al. Reconstructing an inducible homologous recombination system in *Ogataea polymorpha*. Biotechnol J. 2025;20:e70040. 10.1002/biot.70040. 40490980

[bib49] Jiang W, Newell W, Liu J et al. Insights into the methanol utilization capacity of *Y. lipolytica* and improvements through metabolic engineering. Metab Eng. 2025;91:30–43. 10.1016/j.ymben.2025.03.014. 40158687

[bib50] Kaewkrajay C, Chanmethakul T, Limtong S. Assessment of diversity of culturable marine yeasts associated with corals and zoanthids in the gulf of Thailand, south China sea. Microorganisms. 2020;8:474. 10.3390/microorganisms8040474. 32225058 PMC7232451

[bib51] Karbalaei M, Rezaee SA, Farsiani H. *Pichia pastoris*: a highly successful expression system for optimal synthesis of heterologous proteins. J Cell Physiol. 2020;235:5867–81. 10.1002/jcp.29583. 32057111 PMC7228273

[bib52] Khamwachirapithak P, Sae-Tang K, Mhuantong W et al. Optimizing ethanol production in *Saccharomyces cerevisiae* at ambient and elevated temperatures through machine learning-guided combinatorial promoter modifications. ACS Synth Biol. 2023;12:2897–908. 10.1021/acssynbio.3c00199. 37681736 PMC10594650

[bib53] Kotopka BJ, Smolke CD. Model-driven generation of artificial yeast promoters. Nat Commun. 2020;11:2113. 10.1038/s41467-020-15977-4. 32355169 PMC7192914

[bib54] Kurtzman CP, Fell JW, Boekhout T Chapter 1–Definition, classification and nomenclature of the yeasts. In Kurtzman CP, Fell JW, Boekhout T, (eds.), The Yeasts(Fifth Edition). London: Elsevier, 2011,3–5.

[bib55] Kurtzman CP, Robnett CJ. Identification and phylogeny of ascomycetous yeasts from analysis of nuclear large subunit (26S) ribosomal DNA partial sequences. Antonie Van Leeuwenhoek. 1998;73:331–71. 10.1023/A:1001761008817. 9850420

[bib56] Lee ME, DeLoache WC, Cervantes B et al. A highly characterized yeast toolkit for modular, multipart assembly. ACS Synth Biol. 2015;4:975–86. 10.1021/sb500366v. 25871405

[bib57] Levitan A, Gale AN, Dallon EK et al. Comparing the utility of in vivo transposon mutagenesis approaches in yeast species to infer gene essentiality. Curr Genet. 2020;66:1117–34. 10.1007/s00294-020-01096-6. 32681306 PMC7599172

[bib58] Leyton A, Flores L, Mäki-Arvela P et al. Macrocystis pyrifera source of nutrients for the production of carotenoids by a marine yeast *Rhodotorula mucilaginosa*. J Appl Microbiol. 2019;127:1069–79. 10.1111/jam.14362. 31237965

[bib59] Li AH, Yuan FX, Groenewald M et al. Diversity and phylogeny of basidiomycetous yeasts from plant leaves and soil: proposal of two new orders, three new families, eight new genera and one hundred and seven new species. Stud Mycol. 2020;96:17–140. 10.1016/j.simyco.2020.01.002. 32206137 PMC7082220

[bib60] Li B, Liu N, Zhao X. Response mechanisms of *Saccharomyces cerevisiae* to the stress factors present in lignocellulose hydrolysate and strategies for constructing robust strains. Biotechnol Biofuels. 2022a;15:28. 10.1186/s13068-022-02127-9. PMC8922928 35292082

[bib61] Li D, Wang Y, Hu W et al. Application of machine learning classifier to *Candida auris* drug resistance analysis. Front Cell Infect Microbiol. 2021a;11:742062. 10.3389/fcimb.2021.742062. 34722336 PMC8554202

[bib62] Li F, Yuan L, Lu H et al. Deep learning-based *k*_cat_ prediction enables improved enzyme-constrained model reconstruction. Nat Catal. 2022b;5:662–72. 10.1038/s41929-022-00798-z.

[bib63] Li H, Huang L, Zhang Y et al. Production, characterization and immunomodulatory activity of an extracellular polysaccharide from *Rhodotorula mucilaginosa* YL-1 isolated from sea salt field. Mar Drugs. 2020b;18:595. 10.3390/md18120595. 33256151 PMC7760879

[bib64] Li Y, Steenwyk JL, Chang Y et al. A genome-scale phylogeny of the kingdom Fungi. Curr Biol. 2021;31:1653–1665.e5. 10.1016/j.cub.2021.01.074. 33607033 PMC8347878

[bib65] Li Y, Zhai X, Yu W et al. Production of free fatty acids from various carbon sources by *Ogataea polymorpha*. Bioresour Bioprocess. 2022;9:78. 10.1186/s40643-022-00566-8. 38647893 PMC10992350

[bib66] Li Y-W, Yang C-L, Shen Q et al. YALIcloneNHEJ: an efficient modular cloning toolkit for NHEJ integration of multigene pathway and terpenoid production in *Yarrowia lipolytica*. Front Bioeng Biotechnol. 2022c;9:816980. 10.3389/fbioe.2021.816980. 35308823 PMC8924588

[bib67] Li Z, Li C, Cheng P et al. *Rhodotorula mucilaginosa*—alternative sources of natural carotenoids, lipids, and enzymes for industrial use. Heliyon. 2022;8:e11505. 10.1016/j.heliyon.2022.e11505. 36419653 PMC9676536

[bib68] Linder J, Bogard N, Rosenberg AB et al. A generative neural network for maximizing fitness and diversity of synthetic DNA and protein sequences. Cell Syst. 2020;11:49–62.e16. 10.1016/j.cels.2020.05.007. 32711843 PMC8694568

[bib69] Liu Q, Li Y-h, Tao L-f et al. Rational design and characterization of enhanced alcohol-inducible synthetic promoters in *Pichia pastoris*. Appl Environ Microb. 2025;91:e02191–24. 10.1128/aem.02191-24. PMC11784102 39699198

[bib70] Liu X, Xu Y, Luo Y et al. Prokaryotic and eukaryotic promoters identification based on residual network transfer learning. Bioprocess Biosyst Eng. 2022;45:955–67. 10.1007/s00449-022-02716-w. 35279747

[bib71] Lopes M, M MS, R CA et al. *Yarrowia lipolytica* as a biorefinery platform for effluents and solid wastes valorization—challenges and opportunities. Crit Rev Biotechnol. 2022;42:163–83. 10.1080/07388551.2021.1931016. 34157916

[bib72] Lu H, Li F, Yuan L et al. Yeast metabolic innovations emerged via expanded metabolic network and gene positive selection. Mol Syst Biol. 2021;17:e10427. 10.15252/msb.202110427. 34676984 PMC8532513

[bib73] Manfrão-Netto JHC, Gomes AMV, Parachin NS. Advances in using *Hansenula polymorpha* as chassis for recombinant protein production. Front Bioeng Biotechnol. 2019;7:94. 10.3389/fbioe.2019.00094. 31119131 PMC6504786

[bib74] May GE, Akirtava C, Agar-Johnson M et al. Unraveling the influences of sequence and position on yeast uORF activity using massively parallel reporter systems and machine learning. eLife. 2023;12:e69611. 10.7554/eLife.69611. 37227054 PMC10259493

[bib75] Moon SY, An N-Y, Lee JY. Transforming non-conventional yeasts into key players in biotechnology: advances in synthetic biology applications. Front Microbiol. 2025;16:1600187. 10.3389/fmicb.2025.1600187. 40384783 PMC12081427

[bib76] Moreno-Paz S, van der Hoek R, Eliana E et al. Machine learning-guided optimization of p-coumaric acid production in yeast. ACS Synth Biol. 2024;13:1312–22. 10.1021/acssynbio.4c00035. 38545878 PMC11036487

[bib77] Mukherjee M, Blair RH, Wang ZQ.Machine-learning guided elucidation of contribution of individual steps in the mevalonate pathway and construction of a yeast platform strain for terpenoid production. Metab Eng. 2022;74:139–49. 10.1016/j.ymben.2022.10.004. 36341776

[bib78] Mukherjee V, Radecka D, Aerts G et al. Phenotypic landscape of non-conventional yeast species for different stress tolerance traits desirable in bioethanol fermentation. Biotechnol Biofuels. 2017;10:216. 10.1186/s13068-017-0899-5. 28924451 PMC5597992

[bib79] Muzaffar N, Raziq A, Khan MW et al. Recent developments in heterologous expression of cellulases using the *Pichia pastoris* expression system: a comprehensive literature review. Appl Microbiol. 2025;5:22. 10.3390/applmicrobiol5010022.

[bib80] Naranjo-Ortiz MA, Gabaldón T. Fungal evolution: diversity, taxonomy and phylogeny of the Fungi. Biol Rev. 2019;94:2101–37. 10.1111/brv.12550. 31659870 PMC6899921

[bib81] Naseri G, Koffas MAG. Application of combinatorial optimization strategies in synthetic biology. Nat Commun. 2020;11:2446. 10.1038/s41467-020-16175-y. 32415065 PMC7229011

[bib82] Nora LC, Wehrs M, Kim J et al. A toolset of constitutive promoters for metabolic engineering of *Rhodosporidium toruloides*. Microb Cell Fact. 2019;18:117. 10.1186/s12934-019-1167-0. 31255171 PMC6599526

[bib83] Otoupal PB, Ito M, Arkin AP et al. Multiplexed CRISPR-Cas9-based genome editing of *Rhodosporidium toruloides*. mSphere. 2019;4:e00099–19. 10.1128/mSphere.00099-19. 30894433 PMC6429044

[bib84] Pan Y, Yang J, Wu J et al. Current advances of *Pichia pastoris* as cell factories for production of recombinant proteins. Front Microbiol. 2022;13:1059777. 10.3389/fmicb.2022.1059777. 36504810 PMC9730254

[bib85] Park HW, Mason Earles J, Nitin N. Deep learning enabled rapid classification of yeast species in food by imaging of yeast microcolonies. Food Res Int. 2025;201:115604. 10.1016/j.foodres.2024.115604. 39849741

[bib86] Park Y-K, Ledesma-Amaro R. What makes *Yarrowia lipolytica* well suited for industry?. Trends Biotechnol. 2023;41:242–54. 10.1016/j.tibtech.2022.07.006. 35940976

[bib87] Phuengjayaem S, Kingkaew E, Hoondee P et al. Diversity, astaxanthin production, and genomic analysis of *Rhodotorula paludigena* SP9-15. Heliyon. 2023;9:e18280. 10.1016/j.heliyon.2023.e18280. 37539266 PMC10395543

[bib88] Prabhu AA, Gadela R, Bharali B et al. Development of high biomass and lipid yielding medium for newly isolated *Rhodotorula mucilaginosa*. Fuel. 2019;239:874–85. 10.1016/j.fuel.2018.11.088.

[bib89] Radivojević T, Costello Z, Workman K et al. A machine learning automated recommendation tool for synthetic biology. Nat Commun. 2020;11:4879. 10.1038/s41467-020-18008-4. 32978379 PMC7519645

[bib90] Rahbar Saadat Y, Yari Khosroushahi A, Pourghassem Gargari B. Yeast exopolysaccharides and their physiological functions. Folia Microbiol. 2021;66:171–82. 10.1007/s12223-021-00856-2. 33604744

[bib91] Rawat W, Wang Z. Deep convolutional neural networks for image classification: a comprehensive review. Neural Comput. 2017;29:2352–449. 10.1162/NECO_a_00990. 28599112

[bib92] Rodríguez-López M, Bordin N, Lees J et al. Broad functional profiling of fission yeast proteins using phenomics and machine learning. eLife. 2023;12:RP88229. 10.7554/eLife.88229. 37787768 PMC10547477

[bib93] Rosa CA, Lachance M-A, Limtong S et al. Yeasts from tropical forests: biodiversity, ecological interactions, and as sources of bioinnovation. Yeast. 2023;40:511–39. 10.1002/yea.3903. 37921426

[bib94] Roullier-Gall C, David V, Hemmler D et al. Exploring yeast interactions through metabolic profiling. Sci Rep. 2020;10:6073. 10.1038/s41598-020-63182-6. 32269331 PMC7142100

[bib95] Satianpakiranakorn P, Khunnamwong P, Limtong S. Yeast communities of secondary peat swamp forests in Thailand and their antagonistic activities against fungal pathogens cause of plant and postharvest fruit diseases. PLoS One. 2020;15:e0230269. 10.1371/journal.pone.0230269. 32176885 PMC7075701

[bib96] Segal-Kischinevzky C, Romero-Aguilar L, Alcaraz LD et al. Yeasts inhabiting extreme environments and their biotechnological applications. Microorganisms. 2022;10:794. 10.3390/microorganisms10040794. 35456844 PMC9028089

[bib97] Shankarnarayan S, Charlebois D. Machine learning to identify clinically relevant *Candida* yeast species. Med Mycol J. 2024;62:myad134. 10.1093/mmy/myad134. 38130236

[bib98] Shen Q, Yan F, Li Y-W et al. Expansion of YALIcloneHR toolkit for Yarrowia lipolytica combined with Golden Gate and CRISPR technology. Biotechnol Lett. 2024;46:37–46. 10.1007/s10529-023-03444-1. 38064043

[bib99] Sheng J, Feng X. Metabolic engineering of yeast to produce fatty acid-derived biofuels: bottlenecks and solutions. Front Microbiol. 2015;6:554. 10.3389/fmicb.2015.00554. 26106371 PMC4459083

[bib100] Shi C, Wang X, Xiao Z et al. Cloning, characterization and expression analysis of glutathione S-transferase from the Antarctic yeast *Rhodotorula mucilaginosa* AN5. Protein Express Purif. 2020;167:105518. 10.1016/j.pep.2019.105518. 31669543

[bib101] Shimazaki S, Yamada R, Yamamoto Y et al. Building a machine-learning model to predict optimal mevalonate pathway gene expression levels for efficient production of a carotenoid in yeast. Biotechnol J. 2024;19:2300285. 10.1002/biot.202300285. 37953664

[bib102] Siddiq MA, Wittkopp PJ. Mechanisms of regulatory evolution in yeast. Curr Opin Genet Dev. 2022;77:101998. 10.1016/j.gde.2022.101998. 36220001 PMC10117219

[bib103] Spannenkrebs JB, Eiermann A, Zoll T et al. Closing the loop: establishing an autonomous test-learn cycle to optimize induction of bacterial systems using a robotic platform. Front Bioeng Biotechnol. 2025;12:1528224. 10.3389/fbioe.2024.1528224. 39911814 PMC11795046

[bib104] Subash Chandra Bose K, Shah MI, Krishna J et al. Genome-scale metabolic model analysis of *Pichia pastoris* for enhancing the production of S-adenosyl-l-methionine. Bioprocess Biosyst Eng. 2023;46:1471–82. 10.1007/s00449-023-02913-1. 37597025

[bib105] Sun W, Vila-Santa A, Liu N et al. Metabolic engineering of an acid-tolerant yeast strain *Pichia kudriavzevii* for itaconic acid production. Metab Eng Commun. 2020;10:e00124. 10.1016/j.mec.2020.e00124. 32346511 PMC7178482

[bib106] Tan S-I, Liu Z, Tran VG et al. *Issatchenkia orientalis* as a platform organism for cost-effective production of organic acids. Metab Eng. 2025;89:12–21. 10.1016/j.ymben.2025.02.003. 39954846

[bib107] Thorwall S, Schwartz C, Chartron JW et al. Stress-tolerant non-conventional microbes enable next-generation chemical biosynthesis. Nat Chem Biol. 2020;16:113–21. 10.1038/s41589-019-0452-x. 31974527

[bib108] Tobin EE, Collins JH, Marsan CB et al. Omics-driven onboarding of the carotenoid producing red yeast *Xanthophyllomyces dendrorhous* CBS 6938. Appl Microbiol Biotechnol. 2024;108:547. 10.1007/s00253-024-13379-w. 39731599 PMC11682019

[bib109] Vijayakumar VE, Venkataraman K. A systematic review of the potential of *Pichia pastoris* (*Komagataella phaffii*) as an alternative host for biologics production. Mol Biotechnol. 2024;66:1621–39. 10.1007/s12033-023-00803-1. 37400712

[bib110] Wang Y, Li R, Zhao F et al. Metabolic engineering of *Komagataella phaffii* for the efficient utilization of methanol. Microb Cell Fact. 2024;23:198. 10.1186/s12934-024-02475-1. 39014373 PMC11253385

[bib111] Watcharawipas A, Sansatchanon K, Phithakrotchanakoon C et al. Novel carotenogenic gene combinations from red yeasts enhanced lycopene and beta-carotene production in *Saccharomyces cerevisiae* from the low-cost substrate sucrose. FEMS Yeast Res. 2021;21:foab062. 10.1093/femsyr/foab062. 34865010

[bib112] Wefelmeier K, Ebert BE, Blank LM et al. Mix and match: promoters and terminators for tuning gene expression in the methylotrophic yeast *Ogataea polymorpha*. Front Bioeng Biotechnol. 2022;10:876316 . 10.3389/fbioe.2022.876316. 35620471 PMC9127203

[bib113] Wefelmeier K, Schmitz S, Haut AM et al. Engineering the methylotrophic yeast *Ogataea polymorpha* for lactate production from methanol. Front Bioeng Biotechnol. 2023;11:1223726. 10.3389/fbioe.2023.1223726. 37456718 PMC10347679

[bib114] Wefelmeier K, Schmitz S, Kösters BJ et al. Methanol bioconversion into C3, C4, and C5 platform chemicals by the yeast *Ogataea polymorpha*. Microb Cell Fact. 2024;23:8. 10.1186/s12934-023-02283-z. 38172830 PMC10763331

[bib115] Wen Z, Zhang S, Odoh CK et al. *Rhodosporidium toruloides*—A potential red yeast chassis for lipids and beyond. FEMS Yeast Res. 2020;20:foaa038. 10.1093/femsyr/foaa038. 32614407 PMC7334043

[bib116] Wytock TP, Motter AE. Predicting growth rate from gene expression. Proc Natl Acad Sci USA. 2019;116:367–72. 10.1073/pnas.1808080116. 30578321 PMC6329983

[bib117] Xie L, Yu W, Gao J et al. *Ogataea polymorpha* as a next-generation chassis for industrial biotechnology. Trends Biotechnol. 2024;42:1363–78. 10.1016/j.tibtech.2024.03.007. 38622041

[bib118] Yamamoto Y, Yamada R, Matsumoto T et al. Construction of a machine-learning model to predict the optimal gene expression level for efficient production of D-lactic acid in yeast. World J Microbiol Biotechnol. 2023;39:69. 10.1007/s11274-022-03515-x. 36607503

[bib119] Yan C, Yu W, Zhai X et al. Characterizing and engineering promoters for metabolic engineering of *Ogataea polymorpha*. Synth Syst Biotechnol. 2022;7:498–505. 10.1016/j.synbio.2021.12.005. 34977394 PMC8685918

[bib120] Yan Z, Chu W, Sheng Y et al. Integrating deep learning and synthetic biology: a co-design approach for enhancing gene expression via N-terminal coding sequences. ACS Synth Biol. 2024;13:2960–8. 10.1021/acssynbio.4c00371. 39229974

[bib121] Yang Y, Liu G, Chen X et al. High efficiency CRISPR/Cas9 genome editing system with an eliminable episomal sgRNA plasmid in *Pichia pastoris*. Enzyme Microb Technol. 2020;138:109556. 10.1016/j.enzmictec.2020.109556. 32527526

[bib122] Ye M, Gao J, Zhou YJ. Global metabolic rewiring of the nonconventional yeast *Ogataea polymorpha* for biosynthesis of the sesquiterpenoid β-elemene. Metab Eng. 2023;76:225–31. 10.1016/j.ymben.2023.02.008. 36828231

[bib123] Zeng J, Song K, Wang J et al. Characterization and optimization of 5´ untranslated region containing poly-adenine tracts in *Kluyveromyces marxianus* using machine-learning model. Microb Cell Fact. 2024;23:7. 10.1186/s12934-023-02271-3. 38172836 PMC10763412

[bib124] Zhai X, Gao J, Li Y et al. Peroxisomal metabolic coupling improves fatty alcohol production from sole methanol in yeast. Proc Natl Acad Sci USA. 2023;120:e2220816120. 10.1073/pnas.2220816120. 36913588 PMC10041095

[bib125] Zhai X, Ji L, Gao J et al. Characterizing methanol metabolism-related promoters for metabolic engineering of *Ogataea polymorpha*. Appl Microbiol Biotechnol. 2021;105:8761–9. 10.1007/s00253-021-11665-5. 34748038

[bib126] Zhang J, Petersen SD, Radivojevic T et al. Combining mechanistic and machine learning models for predictive engineering and optimization of tryptophan metabolism. Nat Commun. 2020;11:4880. 10.1038/s41467-020-17910-1. 32978375 PMC7519671

[bib127] Zhao M, Yuan Z, Wu L et al. Precise prediction of promoter strength based on a de novo synthetic promoter library coupled with machine learning. ACS Synth Biol. 2022;11:92–102. 10.1021/acssynbio.1c00117. 34927418

[bib128] Zrimec J, Fu X, Muhammad AS et al. Controlling gene expression with deep generative design of regulatory DNA. Nat Commun. 2022;13:5099. 10.1038/s41467-022-32818-8. 36042233 PMC9427793

